# Supplementary material for: Comparative Physiological and Transcriptome Analysis Provide Insights into the Response of Cenococcum geophilum, an Ectomycorrhizal Fungus to Cadmium Stress
Source: J Fungi (Basel). 2022 Jul 12;8(7):724. doi: 10.3390/jof8070724 (PMC9323960; doi:10.3390/jof8070724)
Supplement: Supplementary file 1 [file jof-08-00724-s001.zip › jof-1784126-supplementary.pdf]

**Supplemental Table S1.** Geographic and host information of different strains of *Cenococcum geophilum* used in this paper.

| Isolate No. | Strain number | Isolate location | Host tree species | Isolate time | Isolate source |
|---|---|---|---|---|---|
| C1 | ChCg01 | Yunnan Province, China | *Pinus yunnanensis* | missing | Isolated from ECM |
| J37 | JaCg37 | Numazu Senbonhama, Japan | *Pinus thunbergii* | 20180501 | Isolated from ECM |
| J45 | JaCg45 | Numazu Senbonhama, Japan | *Pinus thunbergii* | 20180507 | Isolated from ECM |
| C78 | ChCg78 | Xinjiang Province, China | *Picea asperata* | 20180902 | Isolated from sclerotium |
| J127 | JaCg127 | Niigata Prefecture, Japan | *Pinus densiflora* | 20180820 | Isolated from sclerotium |
| J202 | JaCg202 | Mount Fuji, Japan | *Salix reinii* | 20181012 | missing |

**Supplemental Table S2.** RT-qPCR gene-ID and its primer sequences.

| Gene-ID | (Sense primer) | (Anti-sense primer) |
|---|---|---|
| gene-K441DRAFT-668187 | 5' CACTGTTCGCTGTTGGAG 3' | 5' GACGGTCGCTGGTAGGAT 3' |
| gene-K441DRAFT-654069 | 5' GGTGACGAGGGTGGTGTT 3' | 5' ACTTGCTCTTGTCGCTGT 3' |
| gene-K441DRAFT-681328 | 5' CATCGTTCGTGCTGTGGC 3' | 5' CAGTTGGTTGTGCGTGGC 3' |
| gene-K441DRAFT-653729 | 5' TTATGACTATCCTCACCCTCG 3' | 5' GCCACTGTGCCTACAACCT 3' |
| gene-K441DRAFT-600384 | 5' TGAGGCGTGTCATAAAGG 3' | 5' AGCAGTTGACGGTGGGTA 3' |
| gene-K441DRAFT-552345 | 5' AAGAGTAATGGCGGTGGC 3' | 5' TCAGCGAGGATTTCAGGT 3' |
| gene-K441DRAFT-660325 | 5' CCAGACCCAGTCATCAGA 3' | 5' AATAGCGTGTTCCTTGTAAT 3' |
| gene-K441DRAFT-603024 | 5' GGAATGCGGAGTGGGTAG 3' | 5' GTAGACGGCGAAGGTGAA 3' |
| gene-K441DRAFT-611738 | 5' TTGAGCAAAGCGTGAAAG 3' | 5' GACCCAGAAGCCACCTAA 3' |
| gene-K441DRAFT-672999 | 5' TTGCCATGTACCCAGACCC 3' | 5' CCTGCTCCAAACCGAACC 3' |

**Supplemental Table S3.** The content of nutrient element (P, K, Na,Ca, Mg, Fe, Al, Mn, Cu and Zn) of 6 strains of *Cenococcum geophilum* under different Cd concentrations

| Experiment strains | Cd concentration (mg/L) | Nutrient element content (mg/L) | | | | | | | | | |
|---|---|---|---|---|---|---|---|---|---|---|---|
| | | P | K | Na | Ca | Mg | Fe | Al | Mn | Cu | Zn |
| C1 | 0 | 14.761±5.746a | 8.692±4.316a | 69.377±35.679a | 8.371±1.196a | 1.373±0.211a | 3.161±0.947a | 0.150±0.065b | 0.018±0.002a | 0.042±0.003a | 0.153±0.018a |
| | 2 | 25.704±3.459a | 15.353±1.473a | 142.009±25.351a | 10.313±2.908a | 2.321±0.251a | 5.155±0.934a | 0.155±0.045a | 0.020±0.001a | 0.049±0.022a | 0.272±0.050a |
| J37 | 0 | 13.958±2.341b | 8.611±1.521b | 88.861±17.260b | 11.975±5.202a | 1.434±0.130a | 4.289±0.402b | 0.110±0.033a | 0.023±0.004a | 0.046±0.018a | 0.395±0.184a |
| | 2 | 25.301±2.661a | 15.962±2.790a | 167.312±15.574a | 11.914±0.540a | 2.845±0.343a | 6.218±0.572a | 0.125±0.039a | 0.023±0.003b | 0.055±0.001a | 0.388±0.103b |
| J45 | 0 | 30.135±4.88a | 24.399±4.653a | 119.763±5.114a | 13.546±1.362a | 3.734±0.686a | 4.567±1.270a | 0.122±0.014a | 0.032±0.001a | 0.072±0.008a | 0.544±0.065a |
| | 2 | 23.643±5.505a | 21.803±5.343a | 107.763±26.869a | 11.375±2.803a | 3.298±0.756a | 3.352±0.617a | 0.146±0.040a | 0.021±0.001a | 0.057±0.012a | 0.317±0.017a |
| C78 | 0 | 11.294±2.572b | 9.073±3.091b | 48.137±6.092ba | 10.915±1.728a | 1.819±0.371a | 3.462±0.564a | 0.103±0.020a | 0.021±0.003b | 0.058±0.008a | 0.459±0.066a |
| | 2 | 23.529±3.908a | 18.585±2.805a | 100.577±14.246a | 12.826±1.295a | 3.569±1.378a | 4.418±0.644a | 0.142±0.058a | 0.021±0.005a | 0.060±0.000a | 0.791±0.158a |
| J127 | 0 | 18.415±1.868a | 9.998±1.022a | 55.725±1.837a | 12.802±0.729a | 3.334±0.219a | 3.254±0.586a | 0.116±0.009b | 0.020±0.003a | 0.061±0.005a | 0.694±0.089a |
| | 2 | 34.711±14.622a | 17.208±8.334a | 103.195±46.419a | 13.431±2.740a | 5.218±2.007a | 3.669±0.708a | 0.121±0.033a | 0.019±0.004a | 0.064±0.008a | 0.627±0.181a |
| J202 | 0 | 40.269±3.136a | 44.422±2.990a | 191.712±26.82a | 12.603±1.826a | 3.947±0.205a | 4.819±0.297a | 0.101±0.036a | 0.032±0.002a | 0.068±0.009a | 0.381±0.032a |
| | 2 | 36.756±5.146a | 45.455±5.301a | 168.314±21.397a | 11.148±0.727a | 3.878±0.373a | 4.801±0.610a | 0.089±0.025a | 0.027±0.002a | 0.059±0.004a | 0.403±0.025a |

**Supplemental Table S4.** Sequencing quality and mapping rates of RNA-seq in different samples

| Samples | Clean reads No. | Clean bases No. | GC Content % | ≥Q30% | Total Reads | Mapped Reads | Uniq Mapped Reads |
|---|---|---|---|---|---|---|---|
| C1CK | 26,176,298 | 7,832,437,067 | 51.43 | 95.18 | 52,352,596 | 76.36% | 75.86% |
| C1Cd | 23,326,949 | 6,975,926,424 | 51.35 | 95.31 | 46,653,898 | 76.82% | 76.31% |
| J45CK | 20,545,811 | 6,151,615,938 | 51.33 | 95.18 | 41,091,622 | 77.47% | 76.92% |
| J45Cd | 22,198,745 | 6,635,335,937 | 52.32 | 94.96 | 44,397,490 | 71.17% | 69.62% |
| C78CK | 21,623,928 | 6,471,659,267 | 51.57 | 95.33 | 43,247,855 | 76.13% | 75.49% |
| C78Cd | 26,298,865 | 7,869,912,151 | 51.62 | 95.35 | 52,597,730 | 74.78% | 74.16% |
| J127CK | 25,900,582 | 7,743,282,090 | 51.79 | 95.19 | 51,801,163 | 53.59% | 53.30% |
| J127Cd | 23,380,036 | 6,997,747,869 | 51.99 | 94.89 | 46,760,072 | 37.81% | 37.71% |

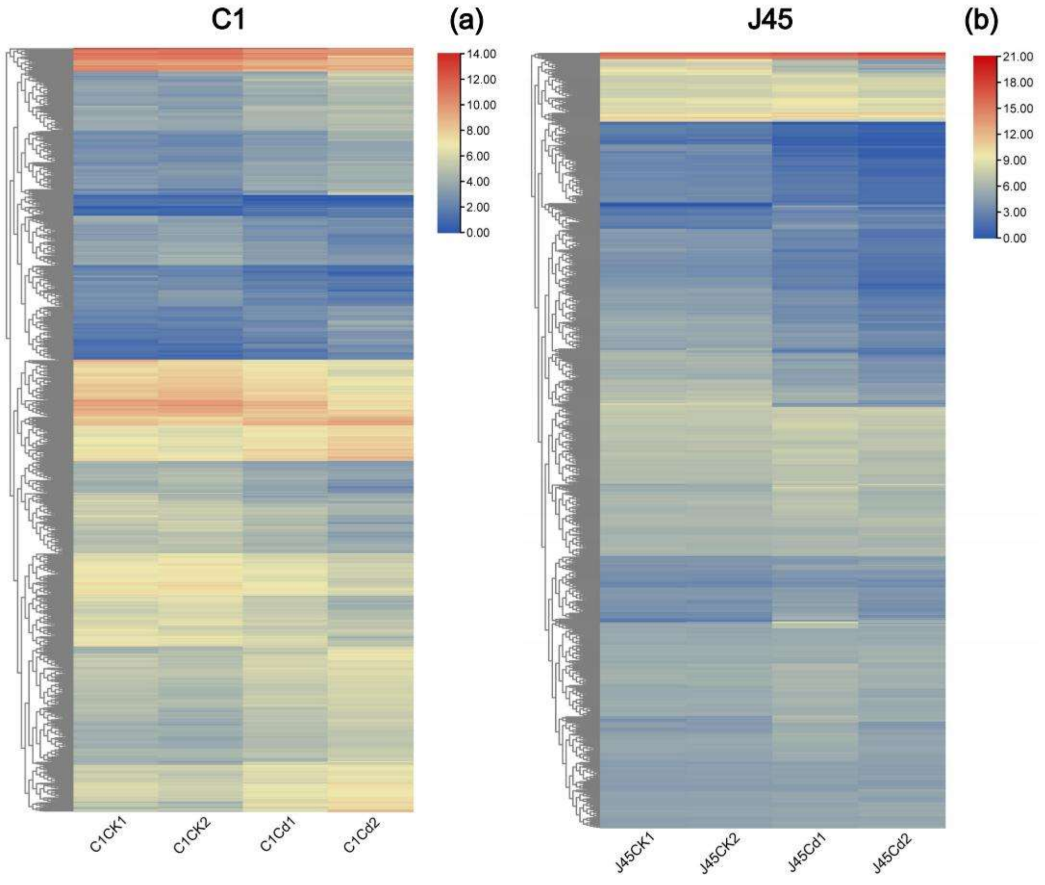
C1
(a)
14.00
12.00
10.00
8.00
6.00
4.00
2.00
0.00
C1CK1
C1CK2
C1Cd1
C1Cd2
J45
(b)
21.00
18.00
15.00
12.00
9.00
6.00
3.00
0.00
J45CK1
J45CK2
J45Cd1
J45Cd2

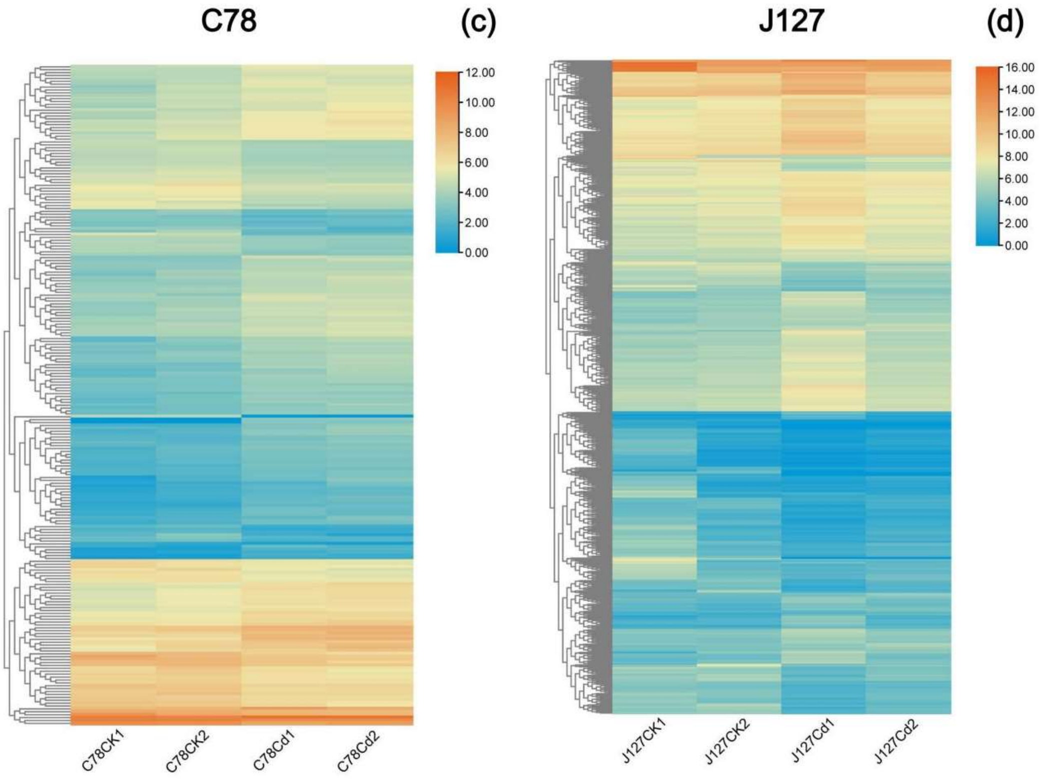


**Supplemental FigureS1.** Heatmap of the DEGs between control and 2 mg/L Cd treatment groups in each *Cenococcum geophilum* strain of C1 (a), J45 (b), C78 (c) and J127 (d). The heat map showed a double hierarchical cluster among DEGs (vertical) and samples (horizontal). The different colors of the heatmap, ranging from blue over white to red represent scaled expression levels of genes with [$\log_2$ (FPKM+1)] across different samples.

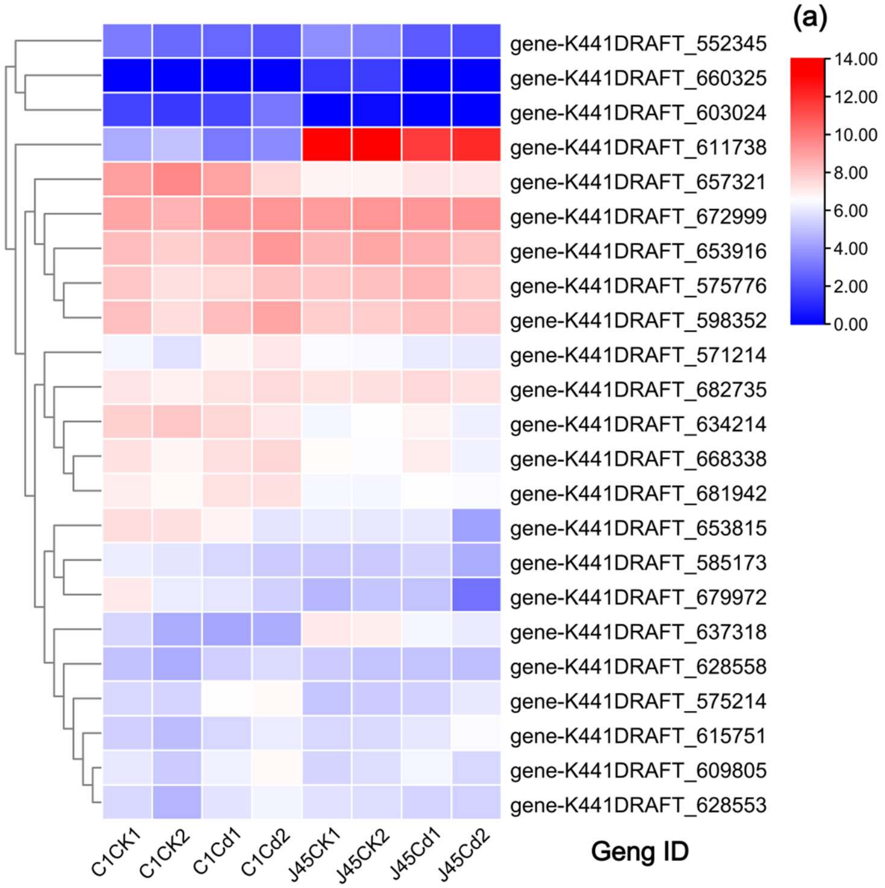
(a)
14.00
12.00
10.00
8.00
6.00
4.00
2.00
0.00
gene-K441DRAFT_552345
gene-K441DRAFT_660325
gene-K441DRAFT_603024
gene-K441DRAFT_611738
gene-K441DRAFT_657321
gene-K441DRAFT_672999
gene-K441DRAFT_653916
gene-K441DRAFT_575776
gene-K441DRAFT_598352
gene-K441DRAFT_571214
gene-K441DRAFT_682735
gene-K441DRAFT_634214
gene-K441DRAFT_668338
gene-K441DRAFT_681942
gene-K441DRAFT_653815
gene-K441DRAFT_585173
gene-K441DRAFT_679972
gene-K441DRAFT_637318
gene-K441DRAFT_628558
gene-K441DRAFT_575214
gene-K441DRAFT_615751
gene-K441DRAFT_609805
gene-K441DRAFT_628553
C1CK1
C1CK2
C1Cd1
C1Cd2
J45CK1
J45CK2
J45Cd1
J45Cd2
Geng ID

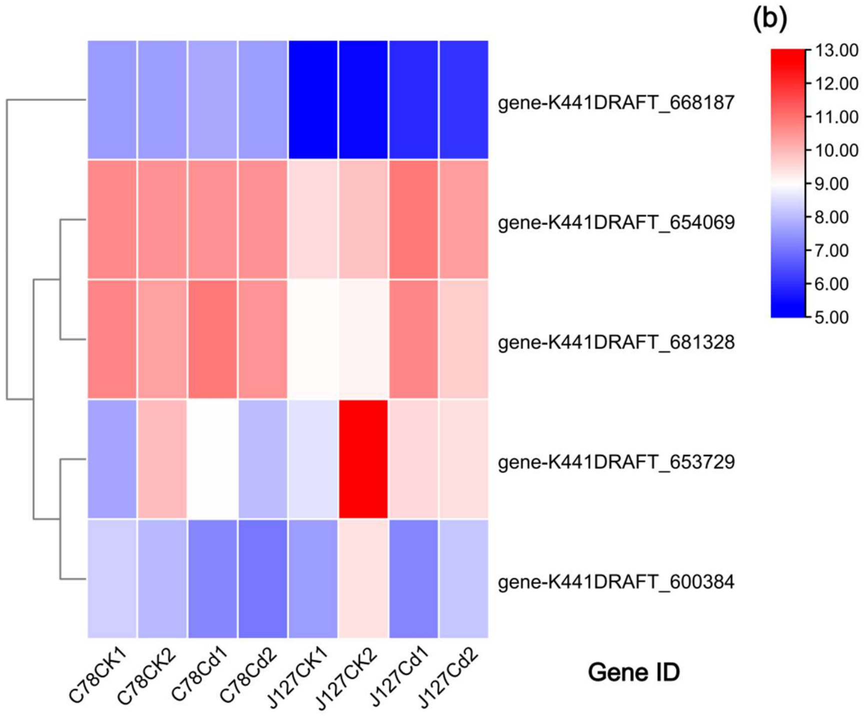


**Supplemental Figure S2.** The heatmap of Kyoto Encyclopedia of Genes and Genomes (KEGG) of the common DEGs between control and 2 mg/L Cd treatment groups in Cd-sensitive group (a) and Cd-tolerant group (b). (a) Heatmap of KEGG of the common genes of the Cd-sensitive group in the common pathways. (b) Heatmap of KEGG of the common genes of the Cd-tolerant group in the pathways of Methane metabolism. Y-axis represents different express genes, respectively. X-axis represents different samples. The heat map showed a double hierarchical cluster among DEGs (vertical) and samples (horizontal). The different colors of the heatmap, ranging from blue over white to red represent scaled expression levels of genes with [$\log_2$ (FPKM+1)] across different samples.

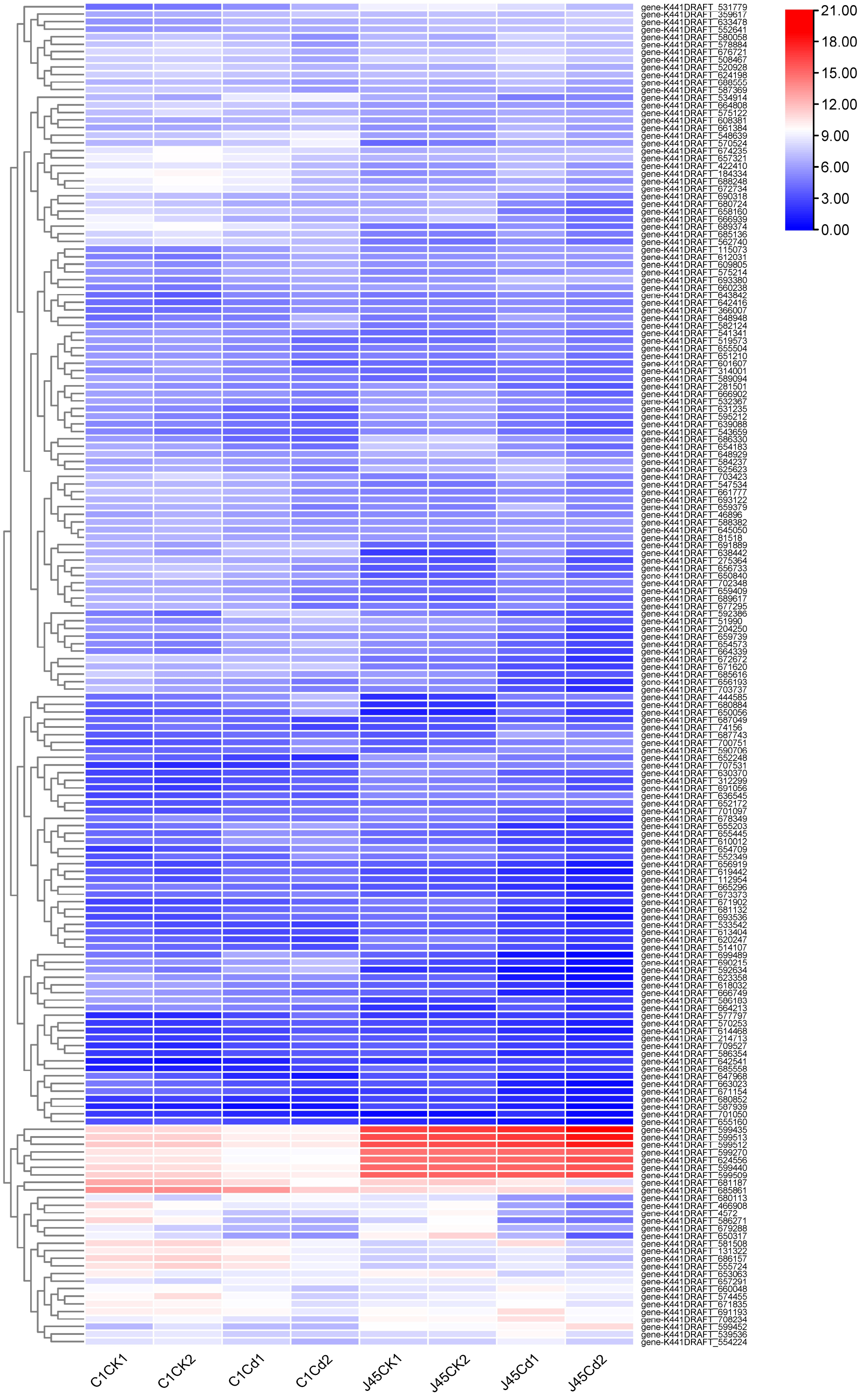
21.00
18.00
15.00
12.00
9.00
6.00
3.00
0.00
C1CK1
C1CK2
C1Cd1
C1Cd2
J45CK1
J45CK2
J45Cd1
J45Cd2

**Supplemental Figure S3.** Heatmap of the common gene for C1 and J45 strains after cadmium treatment. The heat map showed a double hierarchical cluster among DEGs (vertical) and samples (horizontal). The different colors of the heatmap, ranging from blue over white to red represent scaled expression levels of genes with [$\log_2$ (FPKM+1)] across different samples.
